# Assessing the content of a package of SGT-151 sold online

**DOI:** 10.1080/07853890.2021.1897425

**Published:** 2021-09-28

**Authors:** Ana Luzio, Joana Couceiro, Carla Ferreira, Alexandre Quintas

**Affiliations:** a Instituto Universitário Egas Moniz (IUEM), Egas Moniz Cooperativa de Ensino Superior, Caparica, Portugal; b Molecular Pathology & Forensic Biochemistry Laboratory, Centro de Investigação Interdisciplinar Egas Moniz (CiiEM), Egas Moniz Cooperativa de Ensino Superior, Caparica, Portugal; c Laboratório de Ciências Forenses e Psicológicas Egas Moniz, Centro de Investigação Interdisciplinar Egas Moniz (CiiEM), Egas Moniz Cooperativa de Ensino Superior, Caparica, Portugal

## Abstract

**Introduction:**

Synthetic cannabinoids (SCs) are novel psychoactive substances that mimic the effects of cannabis [1–3]. These products were sold *via* online shops and consisted of herbal mixtures sprayed with SCs [3]. Since then, a deluge of chemical variations of SCs has been occurring worldwide due to their synthesis in clandestine laboratories, often based on pharmaceutical research and patents, posing a growing challenge for authorities regarding regulation of these substances [1,3]. More recently, several highly potent compounds have emerged on the drug market, synthesised as stated in the patent application of Bowden and Williamson (“SGT-compounds”). These drugs are characterised by a cumyl substituent, which is attached to an indole, indazole or azaindole structure. One of the first cumyl-derivatives, CUMYL-PEGACLONE, was found in 2016 on the German drug market, being sold under the street name SGT-151 [1,2]. The present study aims to understand what is inside of a SGT-151 package sold in the internet as a ‘research chemical’.

**Materials and methods:**

The cannabinoid identification was based on its mass spectra using the Cayman database (Chemical C. Cayman Spectral Library, vol. v08302018). GC/MS was the technique used to analyse the compound using a MEGA–5 MS capillary column (0.25 µm, 0.32 mm, 30m). Chromatographic analysis was carried out under the following conditions: injection volume 1 µL and splitless injection at 280 °c. The initial oven temperature was 100 °C for 3 min, ramped to 310 °C at a rate of 30 °C/min and held at 310 °C for 10 min. The MS conditions were as follows: ionisation energy was set at 70 eV; acquisition was carried out in a scan mode range of *m/z* 30–450. Helium was used as the carrier gas. For purification, a HPLC/DAD, operated by Clarity software, was used with a reversed-phase column. The mobile phase was a solvent gradient system consisting of (A) 5% 10 mM ammonia format and (B) 95% acetonitrile, optimised to achieve the best resolution. The results were recorded at *λ* = 252 nm.

**Results:**

Figure 1(A) shows the GC/MS chromatogram of SGT-151. It is clear the presence of a peak corresponding to SGT-151 and several other peaks from other compounds. It was not possible to identify the remaining peaks. Figure 1(B) shows a GC/MS chromatogram after purification of the package content. **Discussion and conclusions:** The data gathered in this study shows that SGT-151 contains a significant amount of impurities. This working progress data is the initial step of a bigger project aiming to understand whether there are differences in the toxicity of pure and non-pure SGT-151 on human cell lines, once it is important to understand if the impurities may have any influence on the cytotoxicity, compared to substances in their pure form, since most of the times the substances that are sold on the streets are not 100% pure.

**Figure 1.**
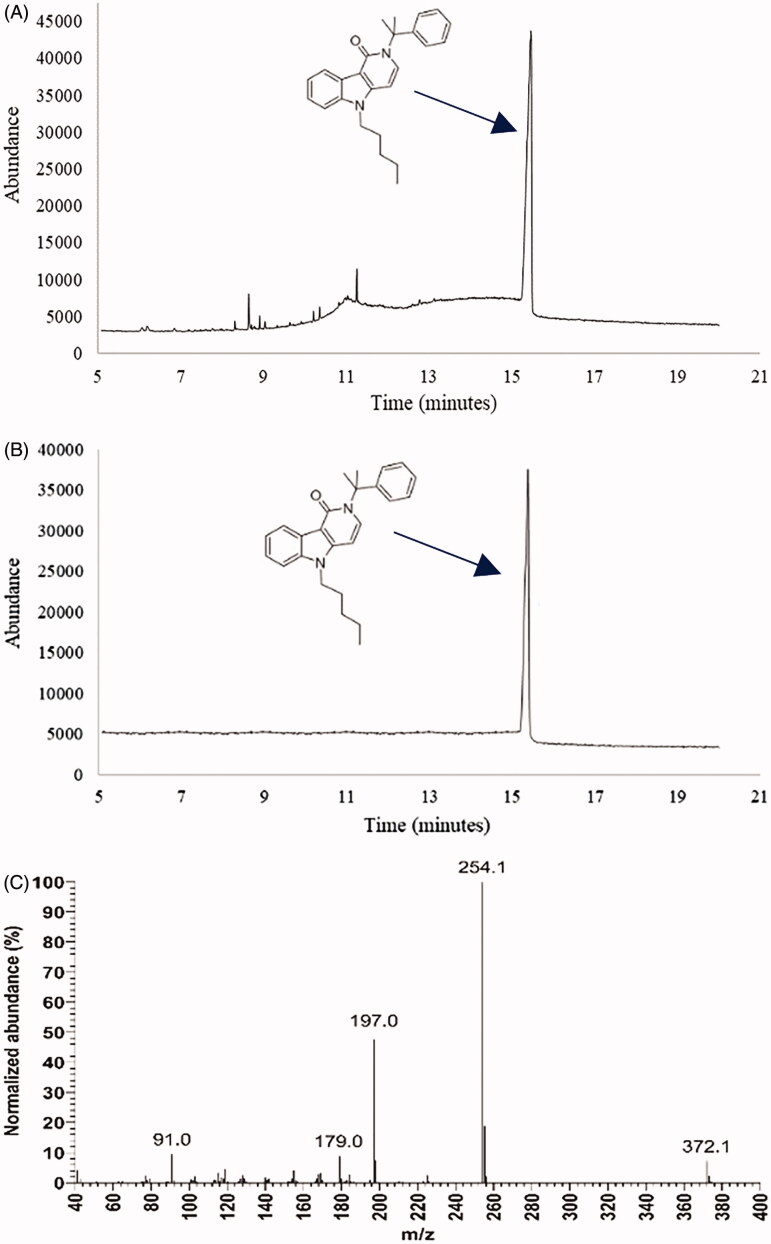
GC/MS analysis: chromatogram before (A) and after (B) purification by HPLC/DAD and mass spectrum (C).
